# Supplementary material for: Gcn2 rescues reprogramming in the absence of Hog1/p38 signaling in C. neoformans during thermal stress
Source: mBio. 2024 Dec 13;16(2):e01762-24. doi: 10.1128/mbio.01762-24 (PMC11796416; doi:10.1128/mbio.01762-24)
Supplement: Supplemental Figures — Figures S1–S6. [file mbio.01762-24-s0001.docx]

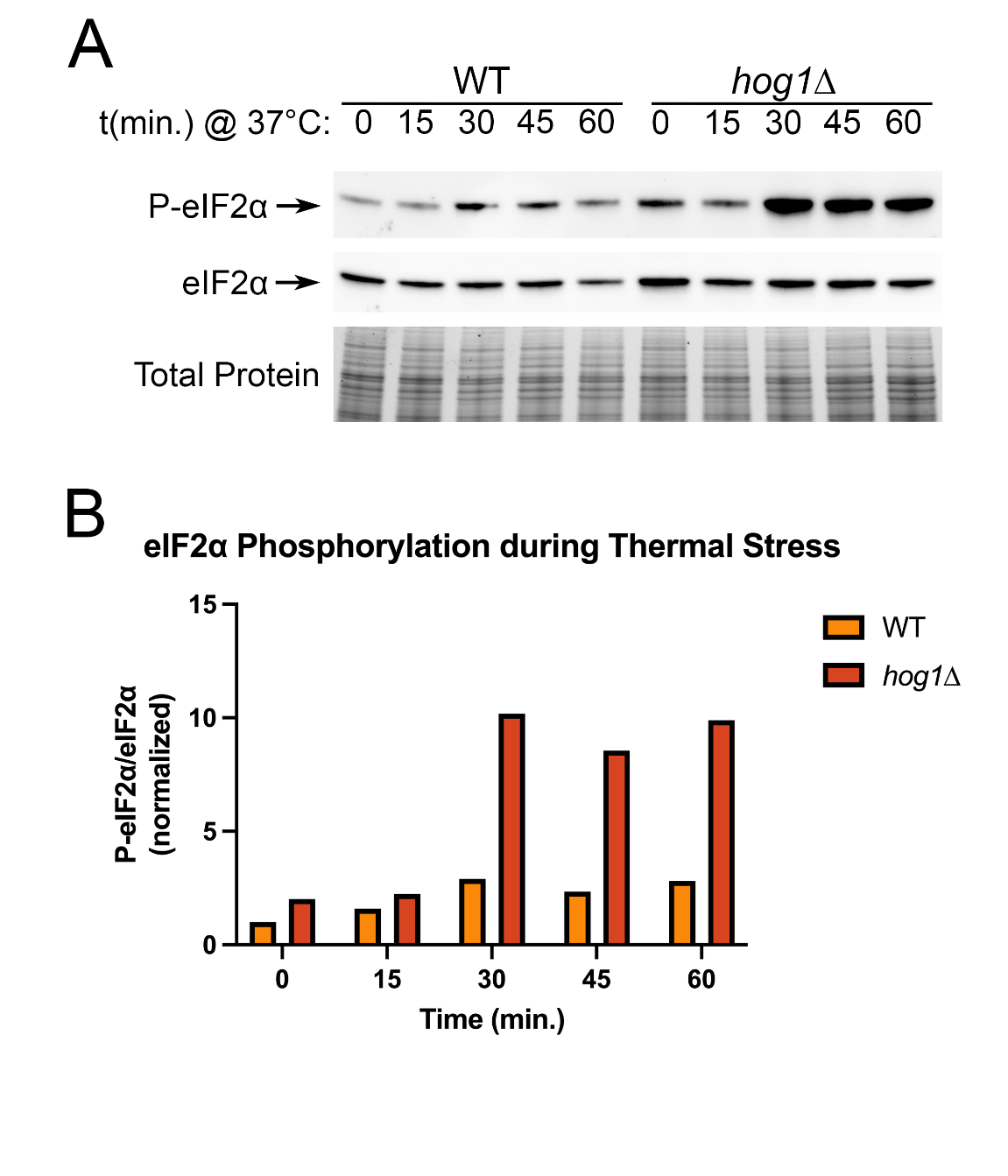


**Fig S1**: **A greater proportion of eIF2α is phosphorylated in *hog1*∆ during thermal stress.** (A) The indicated strains were grown to midlogarithmic phase at 30°C in YPD media, followed by resuspension in pre-warmed 37°C YPD media. Samples were collected at the indicated time points, and protein samples were prepared and probed by western blotting using an antibody against total eIF2α or phosphorylated eIF2α. Total protein is shown as a loading control (*n = 3*). (B) Quantification of signal from panel A, with phosphorylated eIF2α normalized to total eIF2α.


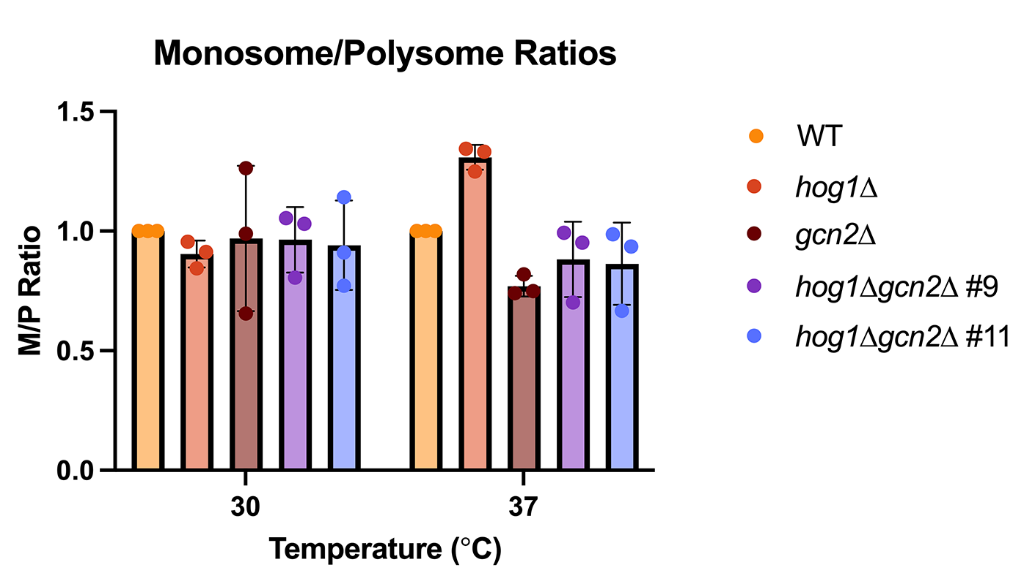


**Fig S2**: **Quantification of polysome profiles during thermal stress.** Polysome profiling was performed as described in Figure 4. The monosome-to-polysome (M/P) ratio was calculated by manually defining the boundaries of these sections of the polysome profile and performing AUC analysis using Prism software. The results for three biological replicates are shown (*n = 3*). Error bars show standard deviation.


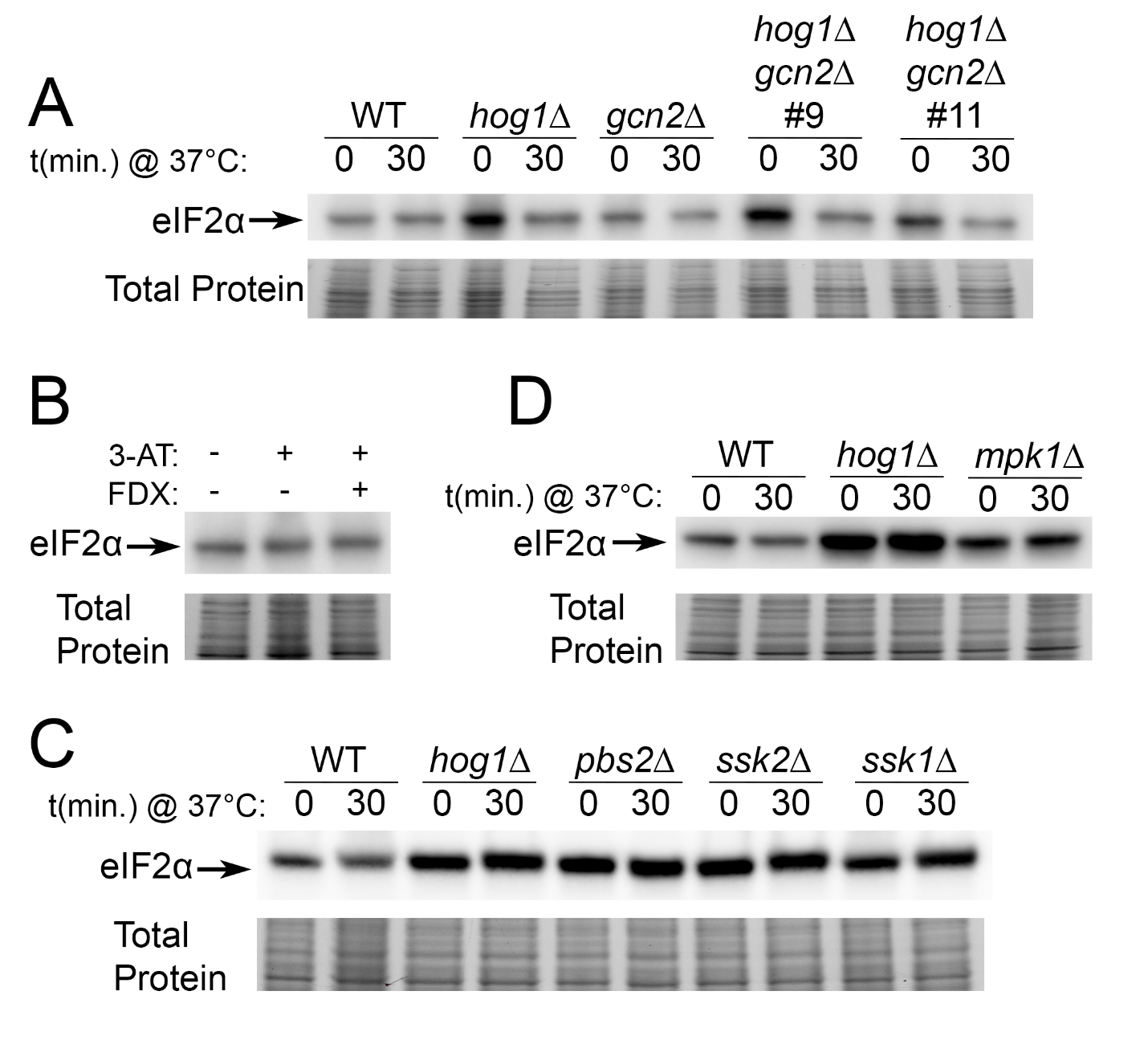


**Figure S3: Total eIF2α signal for *C. neoformans* strains and conditions used in this study.** The indicated strains were grown to midlogarithmic phase in YPD media at 30°C and (A, C-D) resuspended in 37°C media for 30 mins or (B) treated with the indicated drugs for 30 mins. Protein lysates were probed by western blot against total eIF2α with total protein shown as a loading control. A representative blot is shown (*n = 3*).

**
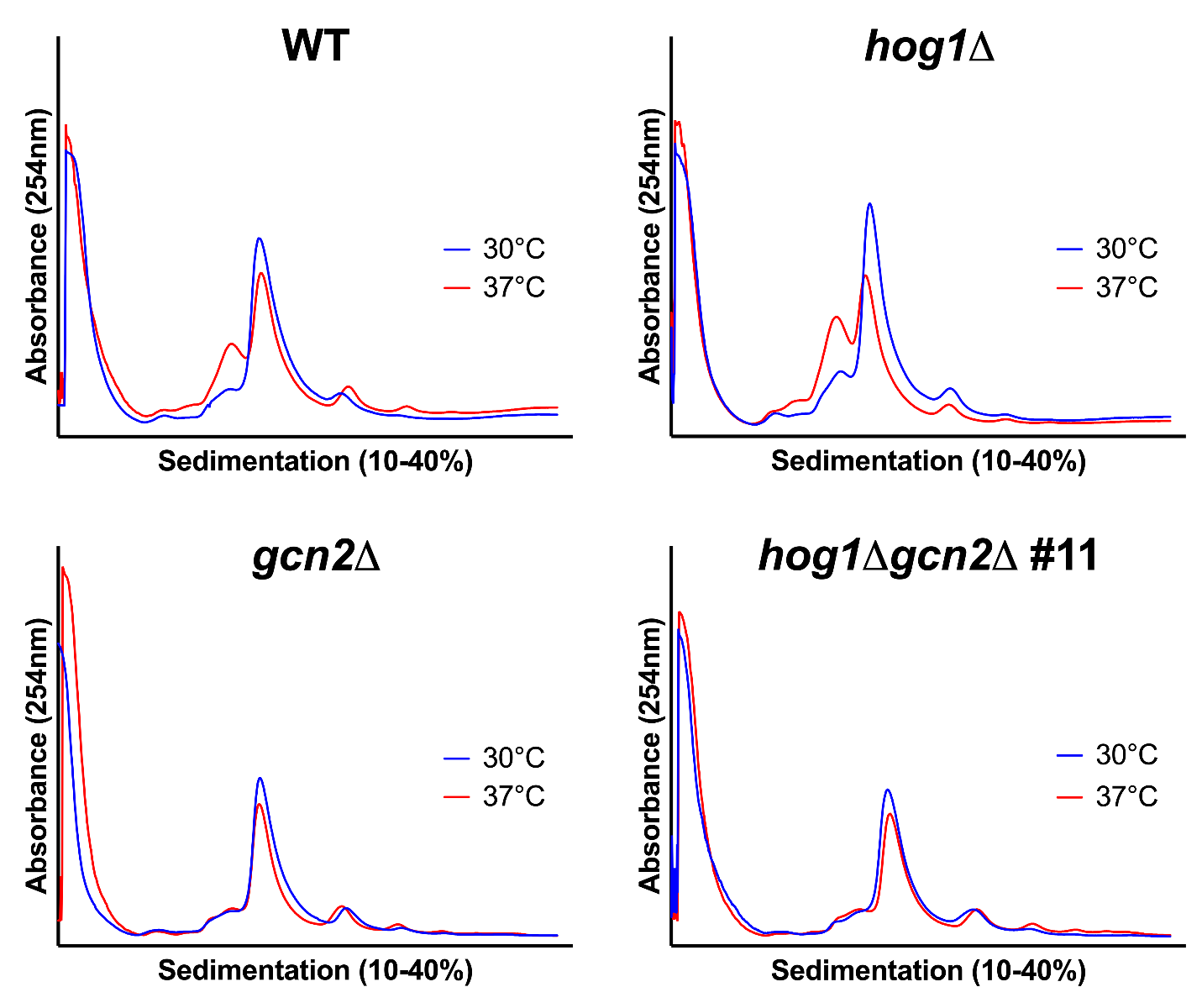
**

**Figure S4: Disome profiles during thermal stress.** The indicated strains were grown to midlogarithmic phase in 30°C YPD media, then resuspended in 37°C media for thirty minutes. Cells were lysed the presence of cycloheximide and equivalent lysate (quantified by OD_280_) was digested with RNase I and centrifuged over a 10-40% sucrose gradient. The resulting A254 signal is shown, with the most prominent peak representing single (non-collided ribosomes) and higher molecular weight peaks corresponding to RNase-resistant complexes of multiple ribosomes.


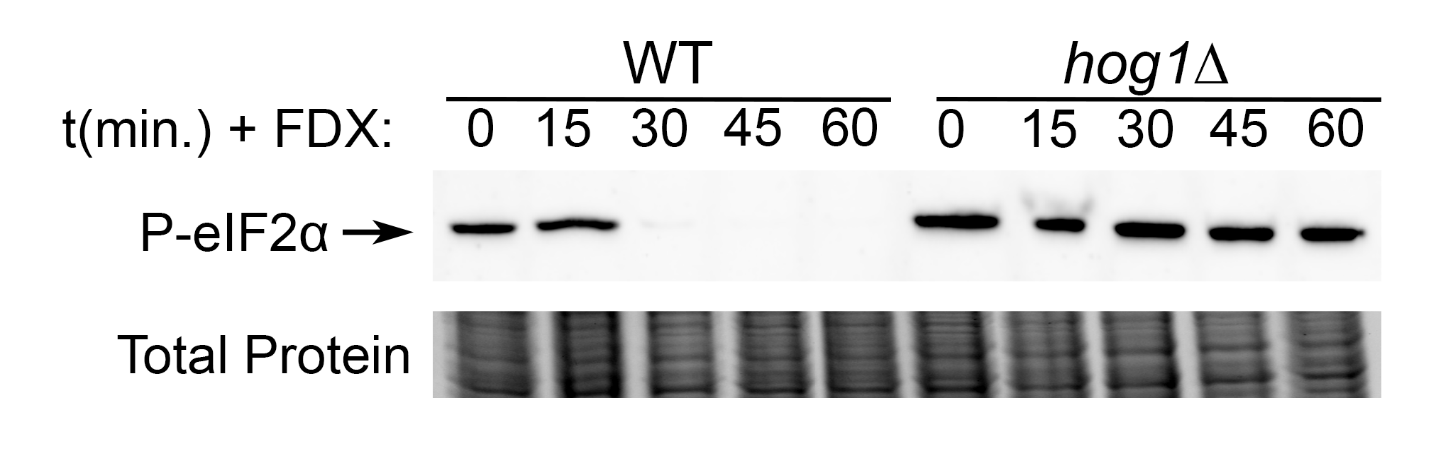


**Figure S5: Fludioxonil alters P-eIF2α in a Hog1-dependent manner.** Cells were grown to mid-logarithmic phase and treated with fludioxonil (FDX) for the indicated amounts of time. Protein lysates were probed for phosphorylated eIF2α by western blot, with total protein shown as a loading control.


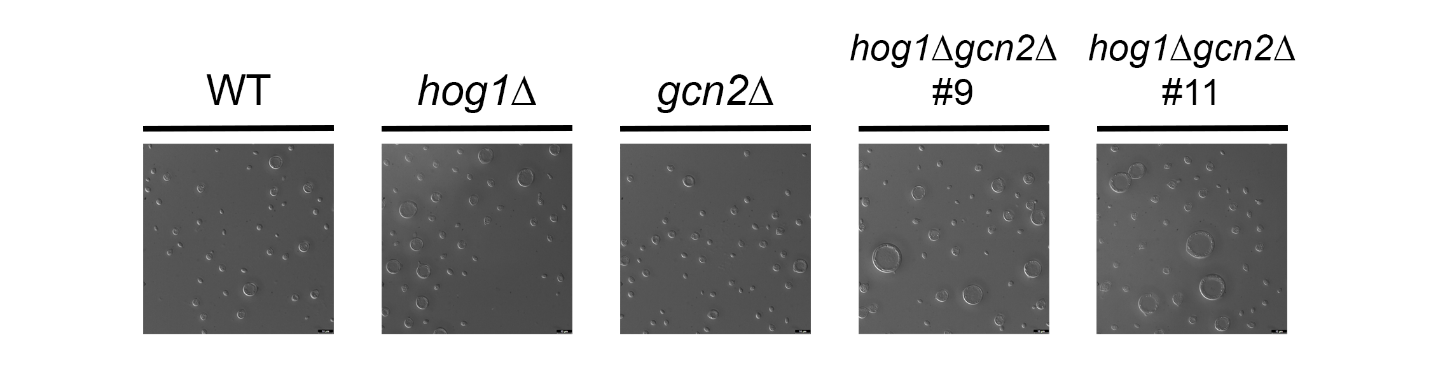


**Figure S6: Gcn2 is dispensible for increased titanization in *hog1*∆ strains.** Shown are representative DIC images of the indicated strains after 48h growth under titan-inducing conditions. Scale bar = 10μM
